# Sudden Development of Anomalous Tumors

**Published:** 1849-03

**Authors:** John A. Cotton

**Affiliations:** Greenwood, Mississippi


ART. V.—*Sudden Development of Anomalous Tumors.* By JOHN A. COTTON, M.D., of Greenwood, Mississippi.

September 14th, 1847, I was requested to see the child of Mr. Henry Darnall, aged about 30 months. At the time of my visit the patient was at play in the yard, and seemed in good health. Upon inquiry, I learned from Mrs. Darnall, that she had that morning discovered a swelling on the child's back that caused her some alarm.

Upon examination, I found on the back of the thorax, to the left of the spine, a tumor of the size and shape of a goose egg divided longitudinally. There was no discoloration of the integuments; no pain on pressure. The circulation was natural; the tongue clean; the alvine evacuations regular and of proper consistency. In fact, all the secretions seemed to be in a healthy condition, and there was no apparent departure from general good health, save the local affection, and that gave no uneasiness to the patient.

The tumor was somewhat resisting, from being pressed upon by the traprezius muscle, and looked very much like those encysted tumors usually denominated wens. My prescription was made more with the view to quiet the apprehensions of the mother than to disperse the tumor, for I really thought the case one requiring no treatment, unless the growth of the tumor should render its removal by the knife necessary.

On the 15th I was again requested to see the child. Another tumor of about half the size of the first and very similar to it had formed immediately below it. It was more superficial than the first, being evidently situated in the subcutaneous cellular tissue; the integuments glided easily over it; it was of a regular oval form, and somewhat moveable. Here was a tumor of the size and nearly the shape of a billiard ball, elevated, of consistent feel, painless, with no mark of inflammation, no general arterial excitement, and, in fact, no perceptible constitutional disturbance, of not more than twelve hours growth.

16th. Was again sent for. The two tumors remained *in statu quo;* but numberless others had formed on almost every part of the body and limbs, varying, in size, from a few lines to an inch, or an inch and a half in diameter. Of these, the most prominent were situated as follows: One on the calf of the left leg; one on the abdomen, to the left of the linea alba, about midway between the pubis and umbilicus; one on the left cheek; one about the center of the forehead; and one on the front of the thorax, to the right of the sternum. All the tumors of any considerable size were situated to the left of the median line, except the two last; but several of the next in size were in the vicinity of the right axilla, and smaller ones were scattered irregularly over the whole body.

After this, there were no more new tumors formed, but the existing crop remained without alteration until the first of November, when they disappeared almost as suddenly as they had formed.

These, to me, most singular formations, were suffered to live out their existence, no treatment for their dispersion having been resorted to. What were they? To one not more accustomed to the investigation of morbid growths than myself, they had every appearance of encysted steatomatous tumors. It gave no pain to handle them, their form could be easily traced, no adhesions existed between them and the integuments, no discoloration of the surface, no fluctuation, no general constitutional disturbance.

Mr. and Mrs. D. do not know that any case, bearing the slightest analogy to the above, ever happened in either branch of the family. I repeat the inquiry, what are these tumors? Are they to be set down to the chapter of accidents, never to be understood, or may it be that a dormant poison yet lingers in the system of the child to develop itself at some future period in the form of a malignant disease?

March, 1848.

